# Analytical dataset on the molecular compositional changes of dissolved organic matter during hyperthermophilic composting

**DOI:** 10.1016/j.dib.2019.104588

**Published:** 2019-10-03

**Authors:** Zhen Yu, Xiaoming Liu, Changya Chen, Hanpeng Liao, Zhi Chen, Shungui Zhou

**Affiliations:** a Guangdong Key Laboratory of Integrated Agro-environmental Pollution Control and Management, Guangdong Institute of Eco-environmental Science & Technology, Guangzhou, 510650, China; b Hunan Provincial Key Laboratory of Fine Ceramics and Powder Materials, School of Materials and Environmental Engineering, Hunan University of Humanities, Science and Technology, Loudi, 417000, China; c Fujian Provincial Key Laboratory of Soil Environmental Health and Regulation, College of Resources and Environment, Fujian Agriculture and Forestry University, Fuzhou, 350002, China

**Keywords:** FT-ICR MS, HTC, DOM transformation, Molecular compound, Molecular formula

## Abstract

The aim of this research work was to determine the molecular compositional changes of dissolved organic matter (DOM) taken from different phases of the hyperthermophilic composting (HTC) process. The DOM samples were extracted by the standard protocol of C18 extraction methodology, and then analyzed by electrospray ionization coupled with Fourier transform ion cyclotron resonance mass spectrometry (ESI FT-ICR MS). The profiles of negative ion mass spectrum and DOM molecular formulas of four compost samples were reported. Data related to the molecular compositional changes of DOM during HTC were also presented. Further interpretation and discussion on these datasets can be found in the related article entitled “Molecular insights into the transformation of dissolved organic matter during hyperthermophilic composting using ESI FT-ICR MS” [1].

**Table:** Specifications Table

Subject	Waste Management and Disposal
Specific subject area	Composting, humification, dissolved organic matter (DOM), electrospray ionization coupled with Fourier transform ion cyclotron resonance mass spectrometry (ESI FT-ICR MS)
Type of data	Graph
How data were acquired	ESI FT-ICR MS: 9.4 T solariX XR FT-ICR MS device (Bruker Daltonik GmbH, Bremen, Germany) equipped with a standard ESI interface
Data format	Raw and analyzed
Experimental factors	Compost samples were collected from different phases of the full-scale hyperthermophilic composting process. For FT-ICR MS analysis, the DOM of compost samples was acidified to pH 2 and extracted by the Sep-pak C18 solid phase extraction cartridges in order to remove any salt.
Experimental features	The DOM was diluted with 10 mL LC-MS grade methanol before the FT-ICR MS analysis. The samples were ionized in negative ion mode using the ESI ion source.
Data source location	Guangzhou Institute of Geochemistry, Chinese Academy of Sciences, Guangzhou, P. R. China.
Data accessibility	Data are accessible with the article.
Related research article	Z. Yu, X. Liu, C. Chen, H. Liao, Z. Chen, S. Zhou, Molecular insights into the transformation of dissolved organic matter during hyperthermophilic composting using ESI FT-ICR MS, Bioresour. Technol. 292 (2019) 122007 [1].

**Table:** 

**Value of the Data** • This data can be useful for understanding the molecular mechanisms on the rapid humification process of HTC. • This data provides evidences that FT-ICR MS is a powerful technique of analyzing the molecular compounds of composted DOM. • The database prompts other researchers to take up similar investigations in DOM transformation during composting by ESI FT-ICR MS.

## 1 Data

**Fig. 1:**
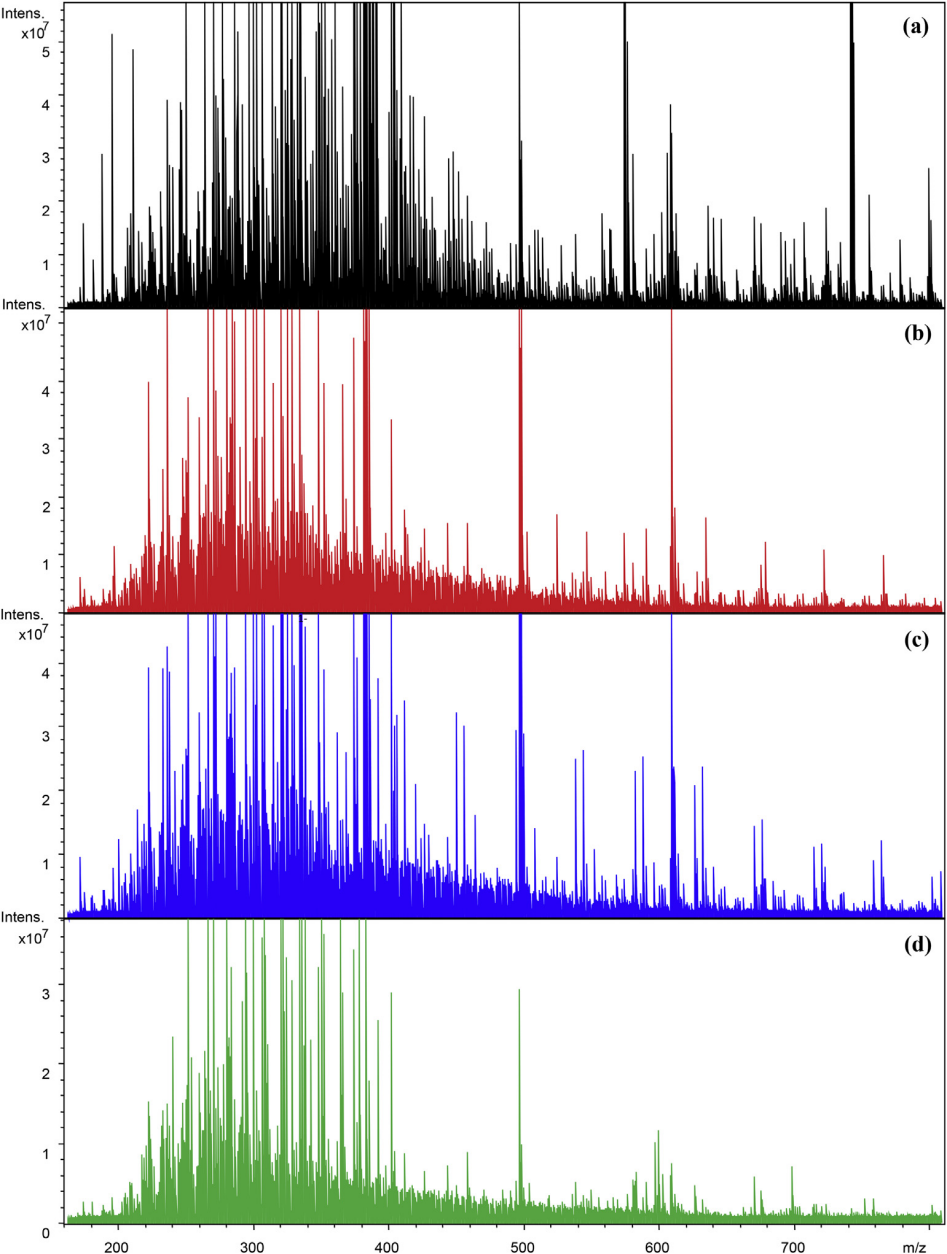
Negative ion mass spectrum of the DOM samples derived from HTC.

Negative ion mass spectra of the C18 extracted DOM samples from the hyperthermophilic composting (HTC) process were presented in Fig. 1. H0 and H45 represent the DOM in composting materials and matured compost, respectively; H9 and H21 denote the DOM of composts collected after the hyperthermophilic and thermophilic phases of HTC, respectively [1]. The mass spectra displayed a similar pattern in the mass range of 150–600 *m/z*, where peaks are mainly distributed for the four DOM samples. The raw data acquired from FT-ICR MS analysis can be found in the supplemental Excel files.

**Fig. 2:**
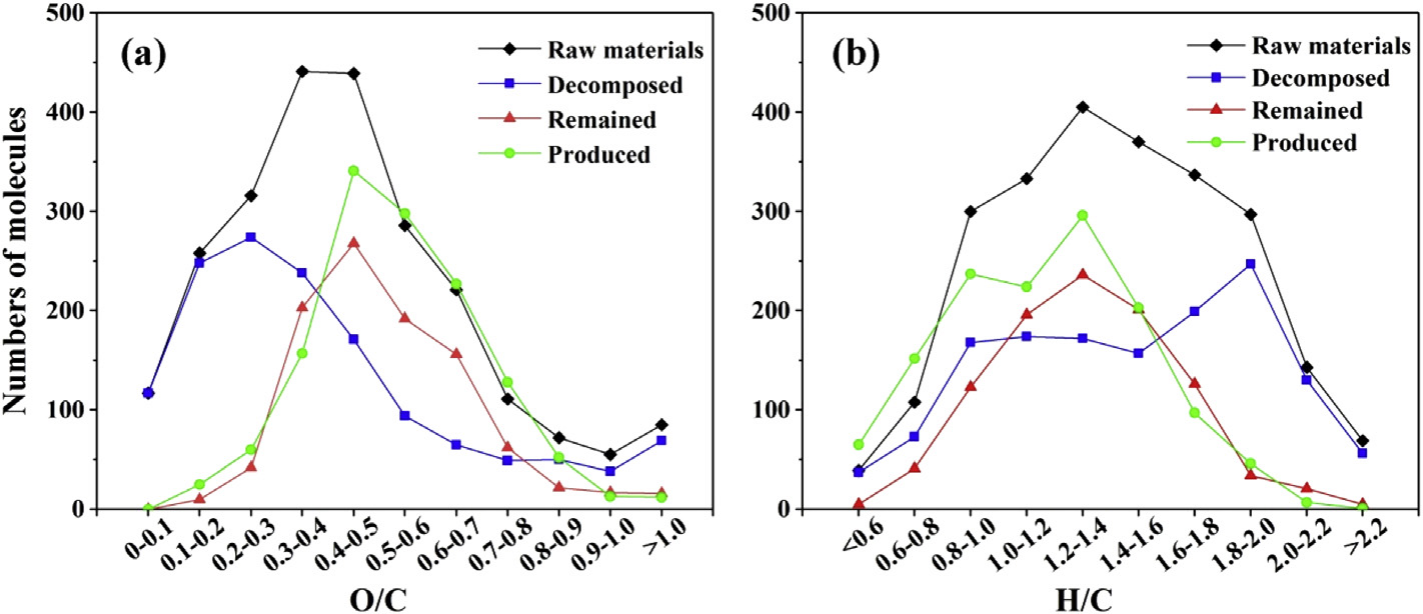
Comparison of the DOM molecular number of O/C and H/C between composting materials and matured compost after HTC.

The quantitative distributions of O/C and H/C of the decomposed, remained, and produced molecules in the DOM of matured compost after HTC were showed in Fig. 2. As a comparison, the number of molecules of O/C and H/C of the DOM in composting materials was also presented.

**Fig. 3:**
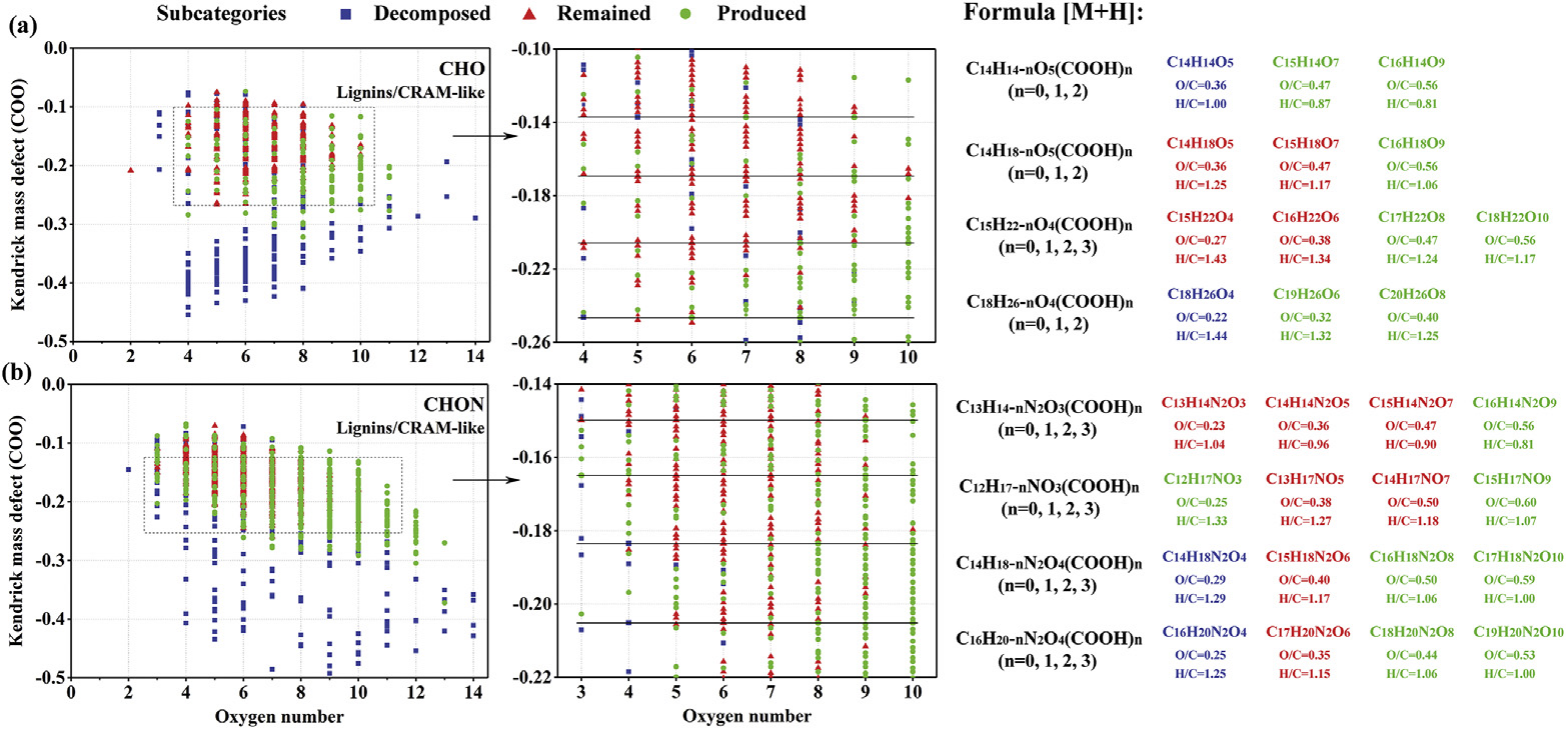
KMD (COO)-number of O in the formulas plots and its scale-expanded segments for lignins/CRAM-like structures of CHO and CHON subcategories in the DOM after HTC.

The data reported in Fig. 3 were used to analyze the transformation of lignins/carboxylic rich alicyclic molecules (CRAM)-like structures of the CHO (containing only C, H, and O) and CHON (containing C, H, O, and N) subcategories during HTC. In the Kendrick mass defect (KMD) plots, the produced lignins/CRAM-like compounds were concentrated in the right upper part (O > 6 and KMD > −0.25), suggesting that the oxidized and unsaturated compounds with more carboxyl groups were mainly formed during HTC. The scale-expanded segments for lignins/CRAM-like structures of the CHO and CHON subcategories and the assigned formulas (as [M−H]^−^ ions) of some randomly selected points in these two segments were also displayed in Fig. 3.

**Fig. 4:**
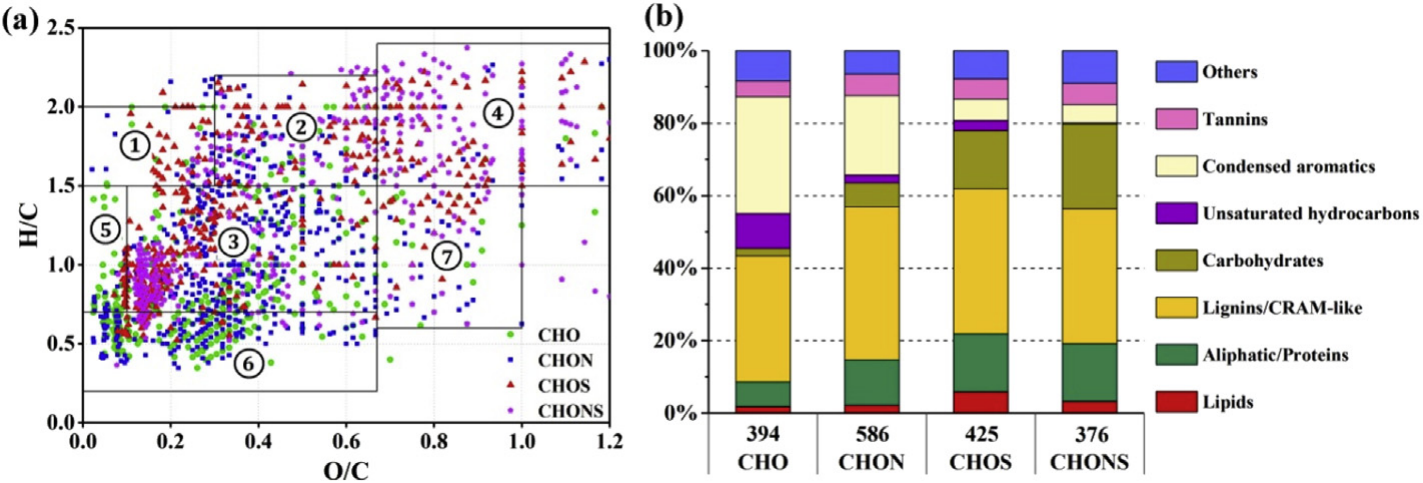
Identification of the DOM molecular formulas as the organic intermediates during HTC.

The data presented in Fig. 4 were used for the identification of DOM molecular compounds as the organic intermediates during HTC. In Fig. 4a, the van Krevelen diagram shows the decomposed DOM compounds in the CHO, CHON, CHOS (containing C, H, O, and S), and CHONS (containing C, H, O, N, and S) subcategories, which were preferentially produced in the hyperthermophilic phase of HTC. Shaded areas with different numbers indicate different biochemical classes of the identified DOM formulas [[2], [3], [4]]: 1, lipids; 2, aliphatic/proteins; 3, lignins/CRAM-like molecules; 4, carbohydrates; 5, unsaturated hydrocarbons; 6, condensed aromatics; and 7, tannins. In Fig. 4b, number percentages of identified DOM compounds in the CHO, CHON, CHOS, and CHONS subcategories were displayed by the bar diagram. The number of formulas identified in each formula is shown on the X-axis of Fig. 4b.

## 2 Experimental design, materials, and methods

### 2.1 DOM samples

The DOM samples were first extracted from four different compost samples collected from the full-scale HTC process of days zero, nine, 21, and 45 as previously described [5,6]. For the FT-ICR MS analysis, the DOM samples were acidified to pH 2 with HCl (pH = 1, MS grade), and then extracted with the Sep-pak C18 solid phase extraction cartridges (500 mg, 6 mL, Waters) in order to remove any salt [4,7]. In brief, the cartridges were rinsed with three volumes of HCl (pH = 2) for the removal of salts, dried with a stream of N_2_, and immediately extracted with 10 mL of methanol (MS grade). Prior to use, the cartridges were activated with three column volumes of methanol and acidified water (pH = 2).

### 2.2 ESI FT-ICR MS analysis

The DOM samples were analyzed by a solariX XR FT-ICR MS (Bruker Daltonik GmbH, Bremen, Germany) equipped with a 9.4 T refrigerated actively shielded superconducting magnet (Bruker Biospin, Wissembourg, France) and the Paracell analyzer cell. The FT-ICR MS conditions were previously reported [7,8]. In brief, the DOM samples were ionized in negative ion mode using the ESI ion source (Bruker Daltonik GmbH, Bremen, Germany). The detection mass range was set to *m/z* 150–1200, and the ion accumulation time was set to 0.6 s. A total of 200 continuous 4 M data FT-ICR transients were co-added to enhance the signal-to-noise ratio and dynamic range. Field blank filters were processed and analyzed following the same procedure to detect possible contamination. The mass spectra were calibrated externally with arginine clusters in the negative ion mode using a linear calibration. The final spectrum was internally recalibrated with typical O_2_ class species peaks using quadratic calibration in DataAnalysis 4.4 (Bruker Daltonics). A typical mass-resolving power >450 000 at *m/z* 319 with <0.3 ppm absolute mass error was achieved.

### 2.3 Data acquisition and processing

The custom software was used to calculate all mathematically possible formulas for all ions with a signal-to-noise ratio above 10 using a mass tolerance of ±1 ppm. The parameters of double bond equivalent (DBE) and modified aromaticity index (AI_mod_) were calculated from the elemental composition C_c_H_h_O_o_N_n_S_s_ [9,10]. The KMD analysis of COO was determined according to the previous studies [3,4]. The stoichiometric ranges used to establish the boundaries of the biochemical classification were previously described [[2], [3], [4]]: lipids (H/C = 1.5–2.0, O/C = 0–0.3), aliphatic/proteins (H/C = 1.5–2.2, O/C = 0.3–0.67), lignins/CRAM-like structures (H/C = 0.7–1.5, O/C = 0.1–0.67), carbohydrates (H/C = 1.5–2.4, O/C = 0.67–1.2), unsaturated hydrocarbons (H/C = 0.7–1.5, O/C = 0–0.1), condensed aromatics (H/C = 0.2–0.7, O/C = 0–0.67), and tannin (H/C = 0.6–1.5, O/C = 0.67–1.0).
